# Combined modality radiation therapy promotes tolerogenic myeloid cell populations and STAT3-related gene expression in head and neck cancer patients

**DOI:** 10.18632/oncotarget.24397

**Published:** 2018-02-02

**Authors:** Sagus Sampath, Haejung Won, Erminia Massarelli, Min Li, Paul Frankel, Nayana Vora, Lalit Vora, Ellie Maghami, Marcin Kortylewski

**Affiliations:** ^1^ Radiation Oncology Beckman Research Institute, City of Hope National Medical Center, Duarte, CA 91010, USA; ^2^ Immuno-Oncology Beckman Research Institute, City of Hope National Medical Center, Duarte, CA 91010, USA; ^3^ Medical Oncology Beckman Research Institute, City of Hope National Medical Center, Duarte, CA 91010, USA; ^4^ Department of Biostatistics, Beckman Research Institute, City of Hope National Medical Center, Duarte, CA 91010, USA; ^5^ Surgery Department Beckman Research Institute, City of Hope National Medical Center, Duarte, CA 91010, USA

**Keywords:** head and neck squamous cell carcinoma, radiation therapy, immunosuppression, IL-6, STAT3

## Abstract

Immunomodulation contributes to the antitumor efficacy of the fractionated radiation therapy (RT). Here, we describe immune effects of RT with concurrent systemic cisplatin or cetuximab treatment of patients with stage III-IV head and neck squamous cell carcinoma (HNSCC). Using longitudinally collected blood samples, we identified significant changes in cytokines/chemokines and immune cell populations compared to immune-related gene expression profiles in peripheral blood mononuclear cells (PBMCs). The 7-week combinatorial RT resulted in gradual elevation of proinflammatory mediators (IFNγ, IL-6, TNFɑ, CCL2), while levels of IL-12, cytokine essential for antitumor immune responses, were decreased. These effects correlated with progressive accumulation of polymorphonuclear myeloid-derived suppressor cells (PMN-MDSC) with detectable activity of STAT3 and PD-L1 expression, underscoring tolerogenic effects of MDSCs. Correspondingly, gene expression analysis of PBMCs harvested after two weeks of combinatorial RT, found upregulation of several immunosuppressive mediators. These included *IL6, IL6R*, *STAT3* and *PDL1*, which could represent IL-6/STAT3-driven tolerogenic signaling, which inhibits T cell and NK activity. Overall, our results suggest that potential immunostimulatory effects of combinatorial RT in HNSCC patients are likely limited by tolerogenic STAT3 signaling and PD-L1 upregulation in myeloid immune cells. Further studies will clarify whether STAT3 targeting could augment RT efficacy and durability of antitumor responses.

## INTRODUCTION

External beam radiation therapy with concurrent chemotherapy or cetuximab, referred to as combined modality therapy (CMT) is a standard treatment for locally advanced head and neck squamous cell carcinoma (HNSCC). The efficacy of CMT is known to be limited not only by intrinsic resistance of cancer cells but also by extrinsic influence of the tumor stroma [1–5]. Despite abundant immunostimulatory signals generated by CMT, spontaneous immune-mediated tumor rejection through so called abscopal effects is rare even after high dose irradiation [6]. The pre-existing immunosuppressive tumor microenvironment and negative feedback regulation of immune responses induced after radiation therapy are likely mitigating the development of systemic tumor resistance [1, 7]. Cell death induced by ionizing radiation or certain types of chemotherapy results in release of mediators, such as cytokines, chemokines or damage-associated molecular patterns (DAMPs), which modulate activity of immune cells recruited into the tumor after treatment [8]. Several tumor-associated, but non-malignant cell populations present in the tumor microenvironment, including endothelial cells [9], fibroblasts [10], tumor-associated macrophages (TAM) [11] and myeloid derived suppressor cells (MDSCs) [12], are implicated in promoting tumor survival and recurrence after local tumor irradiation, specifically in HNSCC [13–17].

Our recent study in preclinical solid tumor models in mice demonstrated that localized tumor irradiation results in release of DAMPs, including Toll-like Receptor 9 (TLR9) ligands, recognized by myeloid immune cells, such as macrophages and MDSCs. TLR9/NF-κB signaling stimulated macrophages to release IL-6, which in turn jump-started proangiogenic and tolerogenic STAT3 signaling leading to tumor recurrence [18]. To verify these observations and gain insights into the potential impact of combinatorial RT on the human immune system, we initiated a proof-of-concept study analyzing changes in soluble mediators and immune-related gene expression patterns in circulating immune cells in patients with advanced HNSCC.

## RESULTS

### Patient characteristics, treatment, and follow-up

From 2015 to 2016, we accrued a total of 15 HNSCC patients with the majority (12 patients) representing human-papilloma virus (HPV)-positive tumors as indicated in the Table 1. Tumor specimens (fine-needle biopsies) were stained for expression of the protein p16/INK4a as a surrogate for HPV status. Thirteen patients received RT with concurrent cisplatin chemotherapy, while two patients received concurrent cetuximab treatment. Two patients demonstrated disease relapse, one of whom was HPV-negative (patient 14). Patient 14 received salvage surgery in the right neck lymph node 3 months following radiation and one year later, developed a second local recurrence in the same location. Patient 15 was p16^+^ presented with large neck nodal disease and received cetuximab with RT. Patient 15 had rapid progression of disease with two months of completing radiation and subsequently died. The remaining 13 patients were alive and free of disease at the time of study submission.

**Table 1:** Patient characteristics

Patient No.	T stage	N stage	No. nodes	Max node size (centimeters)	Chemo	Primary tumor site	HPV status	Disease status
1	2	2c	5	3	q 3 weeks CDDP	tongue base	positive	no recurrence
2	2	2b	4	3.8	q 3 weeks CDDP	tonsil	positive	no recurrence
3	2	2c	5	4.9	q 3 weeks CDDP	tongue base	positive	no recurrence
4	4a	1	1	2.3	q 3 weeks CDDP	tongue base	positive	no recurrence
5	2	2a	1	3	q 3 weeks CDDP	tonsil	positive	no recurrence
6	2	2b	2	2.9	q 3 weeks CDDP	tonsil	positive	no recurrence
7	1	2b	2	2.6	q 3 weeks CDDP	tongue base	positive	no recurrence
8	x	2b	2	3.6	q 3 weeks CDDP	unknown	negative	no recurrence
9	1	2b	2	3.8	Weekly CDDP	tongue base	positive	no recurrence
10	x	2b	2	1.9	Weekly CDDP	unknown	negative	no recurrence
11	4a	2b	3	6	Weekly CDDP	tonsil	positive	no recurrence
12	1	2b	2	2.4	Weekly CDDP	tonsil	positive	no recurrence
13	2	3	7	8	Weekly CDDP	base tongue	positive	no recurrence
14	1	2b	3	2.2	weekly cetuximab	tongue base	negative	Persistent disease in right neck, salvaged with surgery. Second local recurrence in right neck, treated with SBRT. Third recurrence, on anti-PD1 therapy with partial response
15	x	3	6	6.5	weekly cetuximab	unknown	Positive	rapid recurrence, passed away

Abbreviations: HPV, human papilloma virus; CDDP, cisplatin; SBRT, stereotactic body radiation therapy.

### Changes in plasma levels of immune mediators following CMT

**Figure 1:**
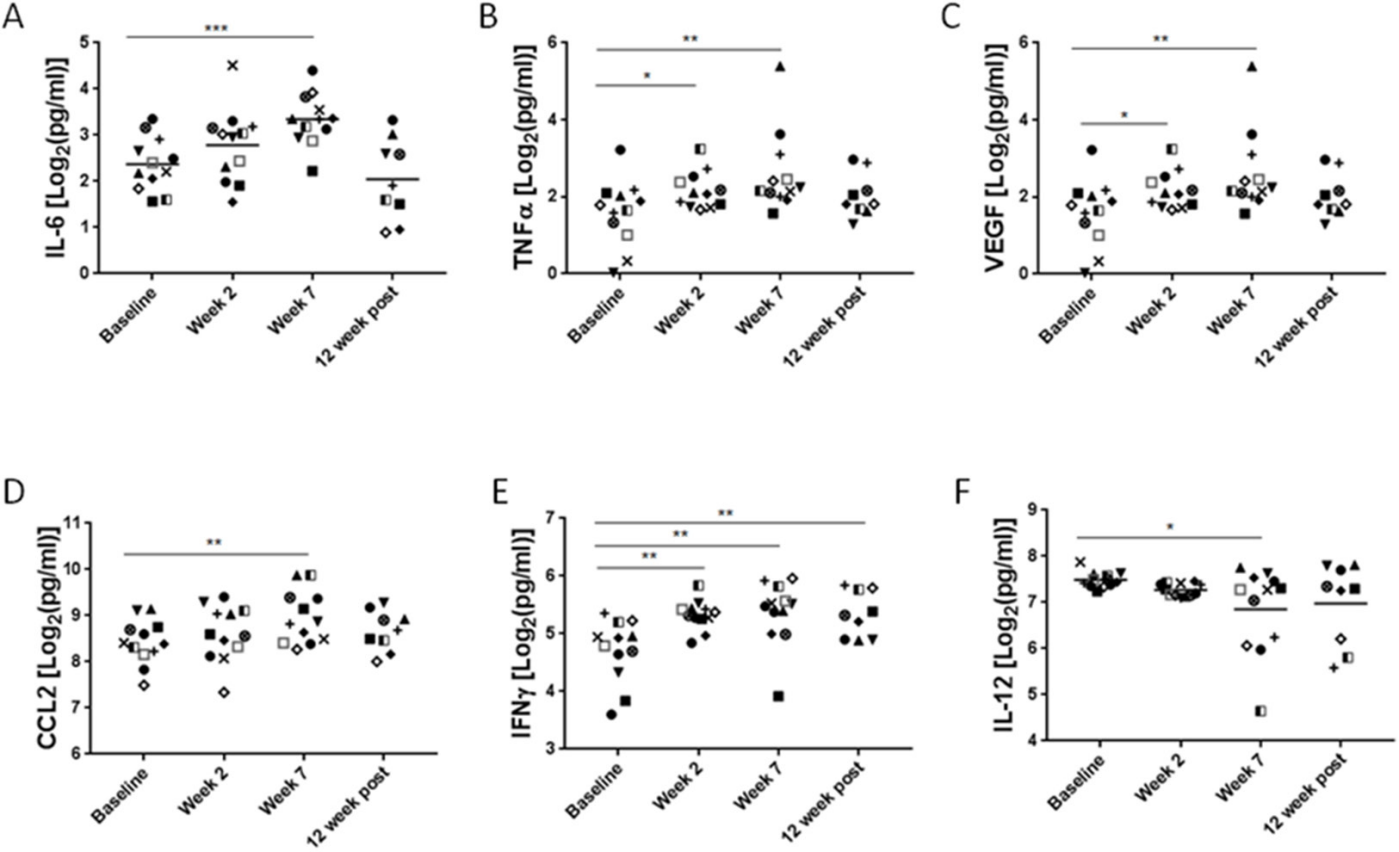
Plasma levels of immune-related mediators in HNSCC patients undergoing CMT Peripheral blood was collected from HPV^+^ HNSCC patients before, during and after CMT treatment completion. Plasma samples were analyzed using for Luminex assay to assess the cytokine/chemokine levels. Different symbols were used to indicate samples from various patients; the HPV-positive patients were shown in color (red, blue and green). Fold changes of IL-6 **(A)**, TNFɑ **(B)**, VEGF **(C)**, CCL2 **(D)**, and IFNγ **(E)**, AND IL-12 **(F)** are shown; *p* values were obtained using paired student *t*-test (baseline *n*=14, ^*^p<0.05, ^**^p<0.005, ^***^p<0.0005).

To assess systemic immunomodulatory effects of the combined chemotherapy and local radiotherapy, we collected plasma samples from patients before, during and after the completion of treatment. Since HPV-mediated HNSCC has a distinct pathogenesis and markedly different clinical outcome, we excluded HPV-negative patients (*n* = 3) from the analysis. The analysis of plasma levels of 30 cytokine and chemokines identified significant changes for two well-known regulators of inflammatory processes and wound healing, namely IL-6 and TNFα. IL-6 increased gradually reaching average 1.5-fold elevation at week 7 of CMT (*p* = 0.0005) and then decreased to the basal level at 12 weeks post-CMT (Figure 1A). The upregulation of TNFα was more transient, with 1.4-fold increase detectable at the second week (*p* = 0.0297). The elevated level remained until the completion of CMT (*p* = 0.0075), and then returned to the baseline level at 12 weeks post-CMT (Figure 1B). Furthermore, we also observed upregulation of VEGF (*p* < 0.05), a strongly proangiogenic and immunosuppressive mediator that increased promptly upon radiation and remained elevated when CMT was completed (Figure 1C). The fourth factor with potential role in cancer radioresistance, a chemokine CCL2/MCP1 critical for monocyte and MDSC recruitment, showed 1.06-fold increase after 7 weeks of CMT (*p* = 0.0017) and then decreased after patients completed therapy (Figure 1D). There were only two positive regulators of antitumor immune responses, IFNγ and IL-12, which were significantly affected by the CMT. While the IFNγ showed moderate 1.14-fold increase during CMT (*p* < 0.0005) and remained elevated thereafter (Figure 1E), the levels of IL-12 decreased over the course of radiation by 10% to the initial level at week 7 during CMT (*p* < 0.05) (Figure 1F). Taken together, these data indicate that continued CMT in HNSCC patients results in release of immune mediators indicative of chronic inflammatory and tolerogenic effect rather than induction of antitumor immune responses.

### Gene expression profiling in circulating immune cells in HNSCC patients

**Figure 2:**
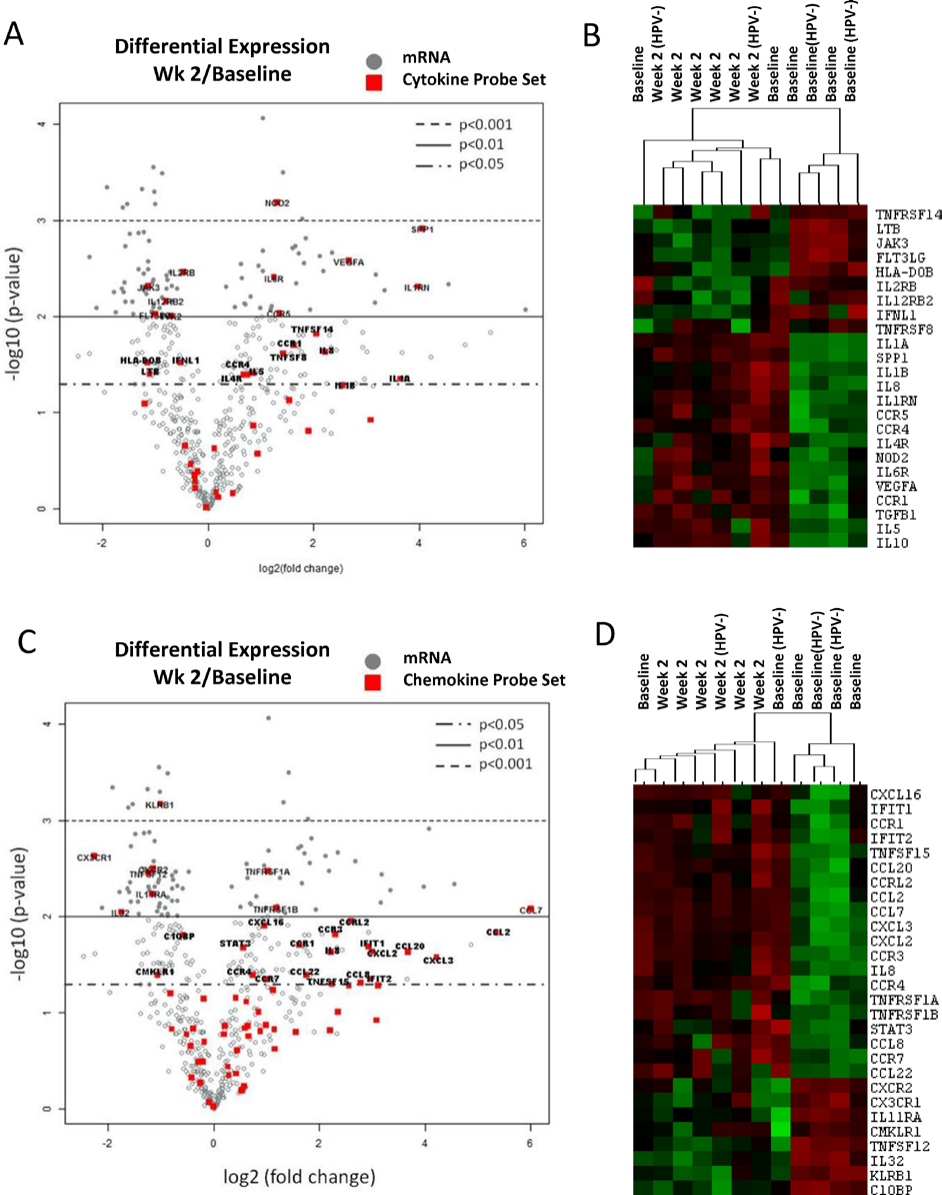
Changes of immune-related gene expression in PBMCs in HNSCC patients after the initial two weeks of CMT PBMC samples were collected from six patients; 4 HPV^+^ and 2 HPV^–^ patients, before and after two weeks of CMT to isolate mRNA. The mRNA samples were analyzed using Nanostring assay on the Human Pancancer Immune panel, followed by the data analysis in the nSolver software. The average fold changes (log2) of the 40 cytokine genes and 69 chemokine genes **(A)** were presented using volcano plots (A and **C**, respectively). The heatmap analysis of selected cytokines and chemokines are shown (**B** and **D**, respectively).

The cytokine/chemokine profiling analysis suggested that CMT regimen could negatively affect the activity of circulating immune cells. Thus, we evaluated gene expression profiles in immune cells isolated from HNSCC patients undergoing CMT. The mRNA was isolated from patients’ PBMCs collected before treatment (baseline) and after two weeks of CMT from the selected 6 patients, combining 4 HPV^+^ patients (pt. 7, 11, 12, and 13) and 2 HPV^–^ patients (pt. 8 and 14) due to limited amount of material. Expression of 770 immune function-related genes was normalized to 20 housekeeping genes (Supplementary Table 6) and analyzed using the Nanostring gene expression assay using nSolver software. The statistical analysis indicated that 206 genes were differentially expressed (*p* < 0.05) at week 2 radiation compared to the baseline. Functional gene set analysis showed that expression of 23 out of 41 tested genes involved in cytokine signaling were significantly changed (*p* < 0.05) after two weeks of CMT (Figure 2A and Supplementary Table 2). The heatmap analysis showed clustering of cytokine gene expression profiles between baseline and samples at week 2 for the majority of patients (Figure 2B). Consistently with the plasma analytes data (Figure 1), we found significant elevation of *VEGFA* expression in PBMCs from treated patients. IL-12 receptor beta 2 subunit (*IL12RB2*) was significantly decreased, corresponding to the reduced plasma levels of IL-12. In contrast, the expression of Th2-promoting mediators, such as *IL5, IL10, TGFb1* and *IL4R*, were significantly increased during CMT. In addition, we found significant increase in levels of IL-1 family cytokine genes, *IL1A*, and *IL1B*, which are well known upstream regulators of *IL6* expression (Figure 2A, 2B and Supplementary Table 2). Beyond cytokine and growth factor genes, we found significant alterations in 28 out of 69 chemokine genes (*p* < 0.05) after two weeks of radiation (Figure 2C and Supplementary Table 3). The CMT increased expression of chemokines or chemokine receptors involved in recruitment of monocytes, macrophages, neutrophils and lymphocytes, such as *CCL7, CCL2, CXCL2, CCL20, CXCL3, CCR7*, and *CCL8*. Finally, we assessed how the above discussed cytokine/chemokine gene profiles match to the expression of genes involved in T cell and NK activity. In fact, the heatmap analysis of cytokine gene set showed strong clustering of the majority of baseline samples (*r* = 0.43) and week 2 samples (*r* = 0.56) and significant decrease in cytotoxicity-related target genes such as *CXCR3, CXCR2, IL32* and *KLRB1* (Figure 2D and Supplementary Table 3). Taken together, the gene expression analysis results suggests that local CMT in tumor affects global gene expression in circulating immune cells, which may alter the systemic immune profile in HNSCC patients [19].

### Increased IL-6-STAT3 signaling in myeloid cells during CMT

**Figure 3:**
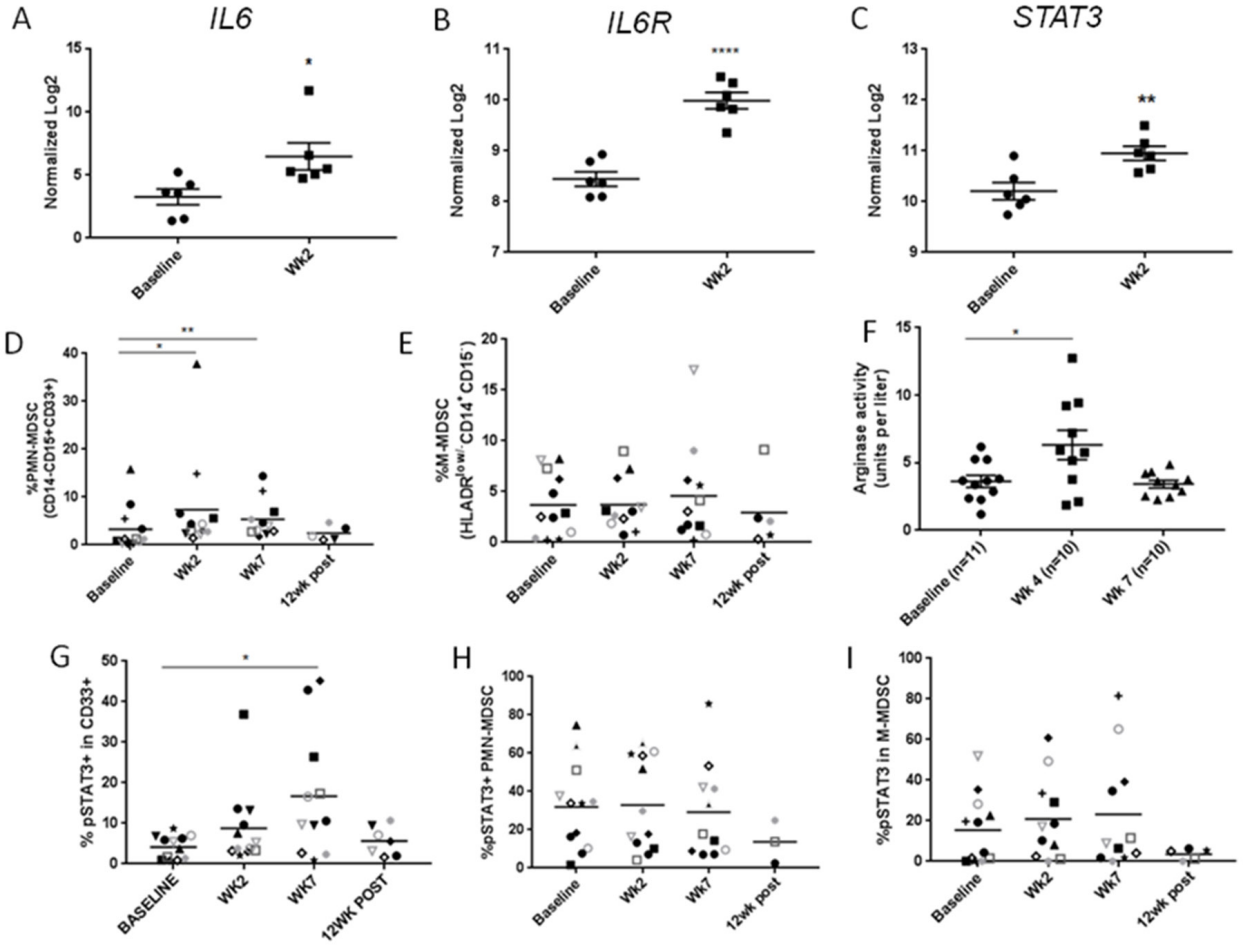
The accumulation of PMN-MDSCs with STAT3 activity in HNSCC patients after CMT The results of Nanostring analysis detecting levels of mRNA expression of *IL6* **(A)**, *IL6R* **(B)**, *STAT3* **(C)** in HNSCC patients’ PBMC samples at baseline and after two weeks of CMT (*n*=6). Percentage of PMN-MDSC (CD14^-^CD15^+^CD33^+^) **(D)** and M-MDSC (HLA-DR^low/-^CD14^+^CD15^-^) **(E)** in HNSCC patients’ PBMCs were analyzed using multcolor flow cytometry. **(F)** Arginase activity was measured in plasma samples drawn before CMT (baseline), at week 4, and at week 7. Frequencies of pSTAT3^+^ expression was quantified in CD33^+^ myeloid population **(G)**, PMN-MDSC **(H)**, and M-MDSC **(I)** using flow cytometry. Paired student *t*-test; ^*^p<0.05, ^**^p<0.005, ^***^p<0.0005.

Consistently with the increased level of IL-6 in plasma during CMT, mRNA expression levels of *IL6*, *IL6ST* and *STAT3* as a downstream signaling molecule were significantly increased in patients’ PBMCs two weeks after initiation of CMT when compared to baseline (Figure 3A–3C). IL-6 is widely recognized for its crucial role in cancer promoting inflammation, which relies on STAT3 as both survival and tolerogenic signaling [20, 21]. IL-6/STAT3 signaling recruits and modulates function of tumor-infiltrating myeloid cell populations in cancer patients [21, 22]. Therefore, we assessed whether increased serum IL-6 level during CMT is reflected by increased percentage of circulating myeloid cells/MDSC as well as STAT3 activation in HPV^+^ patients. In fact, the percentage of circulating PMN-MDSCs increased significantly over baseline at weeks 2 and 7 during CMT (Figure 3D), but the numbers of M-MDSCs did not change (Figure 3E). Noteworthy, these changes correlated with transient elevation of plasma levels of arginase activity, which is a potently immunosuppressive enzyme inhibiting T cell activity that can be expressed by HNSCC-associated PMN-MDSC in STAT3-dependent manner [23] (Figure 3F). STAT3 activity, detected by tyrosine 705-phosphorylation, was observed in the significant percentage of total CD33^+^ myeloid cells at week 7 and decreased back to the baseline level at 12 week post CMT (Figure 3G). When we examined the specific MDSC subsets, we found that STAT3 was consistently activated in about 30% of PMN-MDSC and 20% of M-MDSC (Figure 3H and 3I, respectively), but the weak increase during CMT was not significant likely due to various kinetics of STAT3 elevation in different patients. These results suggest that CMT can activate potentially tolerogenic IL-6/STAT3 signaling in circulating myeloid populations.

### Decreased expression of genes for T cell and NK cell function

**Figure 4:**
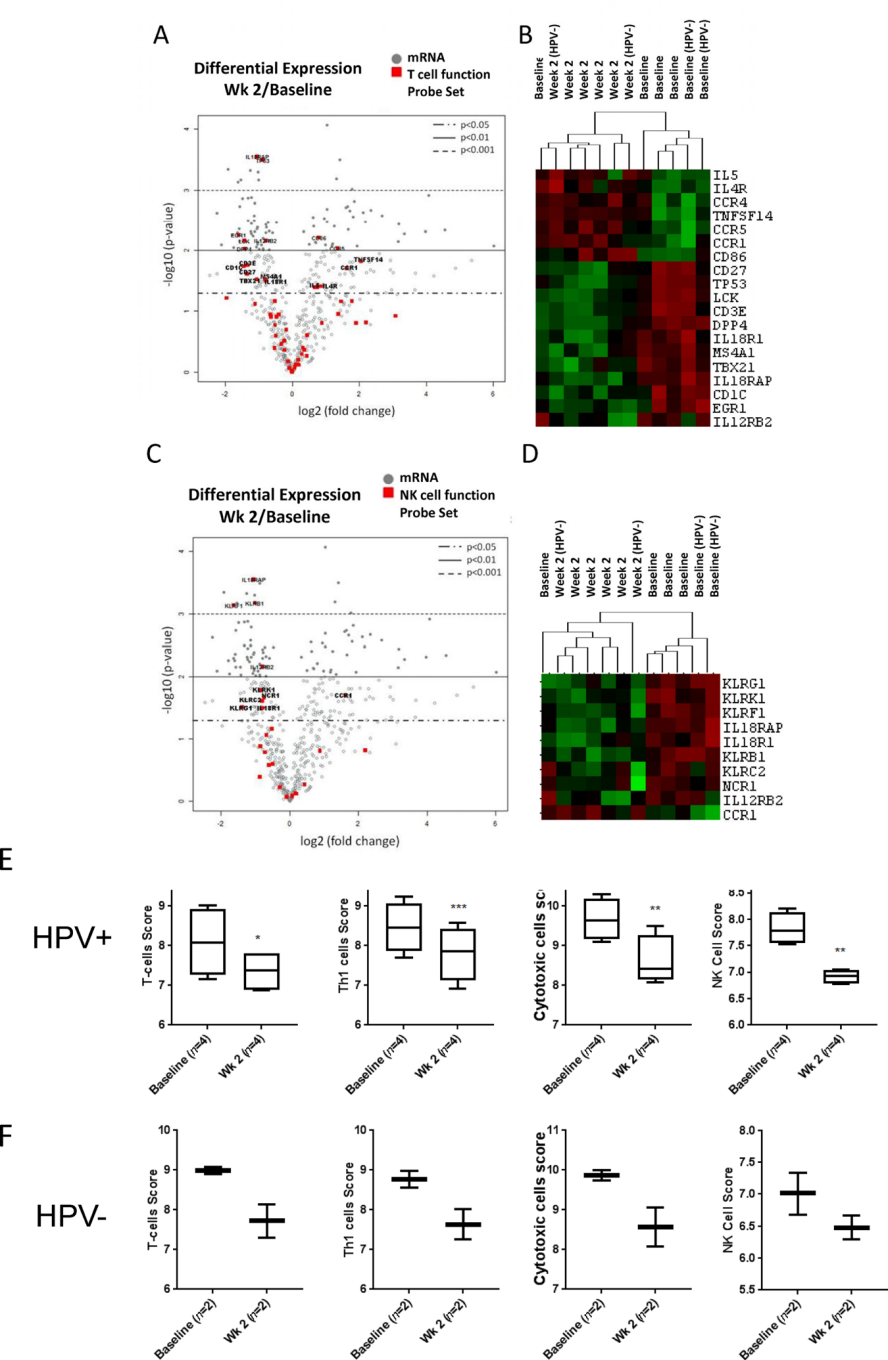
Decreased expression of genes for T cell and NK cell function The results of Nanostring analysis detecting levels of mRNA expression of T cell and NK cell activity-regulating genes. The average fold changes (log2) of genes in T cell function panel **(A)** and NK cell function panel **(C)** after two weeks of CMT are presented using volcano plot (*n*=6). The heatmaps of selected T cell-related **(B)** and NK cell-related genes **(D)** are shown. **(E-F)** Box-and-whisker plots indicating gene expression-based cellular scores for T-cells, Th1 cells, cytotoxic cells, and NK cells analyzed separately in HPV^+^ (E) and HPV^–^ (F) patients.

The activation of IL6/STAT3 signaling suggested by our cytokine analysis and immunophenotyping studies could translate into reduced activity of cytotoxic immune cells, such as T lymphocytes and NK cells. Thus, we compared mRNA expression of a subset of genes involved in the regulation of T cell receptor (TCR) signaling and cytotoxicity in total PBMC before and after first two weeks of CMT. We found that 19 out of 56 genes related to T cell functions were differentially expressed (*p* < 0.05) during CMT, including reduced expression of *CD3e* potentially indicating reduced T cell expansion (Figure 4A–4B and Supplementary Table 4). In addition, we found that expression of *LCK*, a kinase critical for mediating TCR signaling, was significantly reduced during CMT. Expression level of *TBX21* (T-bet), a master regulator of Th1 cell differentiation and cytokine production, was significantly reduced during CMT as well. In contrast, we found the expression levels of Th2-related mediators, such as *IL5* and *IL4R*, were elevated. Since NK cell plays important roles in cytotoxic response against cancer cells, we also evaluate expression of subset of genes regulating NK cell functions (Figure 4C–4D and Supplementary Table 5). The expression of 9 out of 27 NK function-related genes was significantly downregulated, and only *CCR1* showed increased expression. The heatmap analysis of T cell- and NK cell-related genes (*p* < 0.05) with significantly altered expression showed separated clustering of baseline and week 2 samples, except for one patient. Consistently, the predictive gene signatures for the presence/activity of T cells, Th1 cells, cytotoxic cells and NK cells were all significantly reduced at the second week of CMT, both in HPV^+^ and HPV^–^ patients (Figure 4E and 4F, respectively). These results suggest that CMT regimen may be insufficient for generation of the strong Th1 antitumor immunity and immune cell cytotoxicity.

### CMT is associated with increased immune checkpoint control

**Figure 5:**
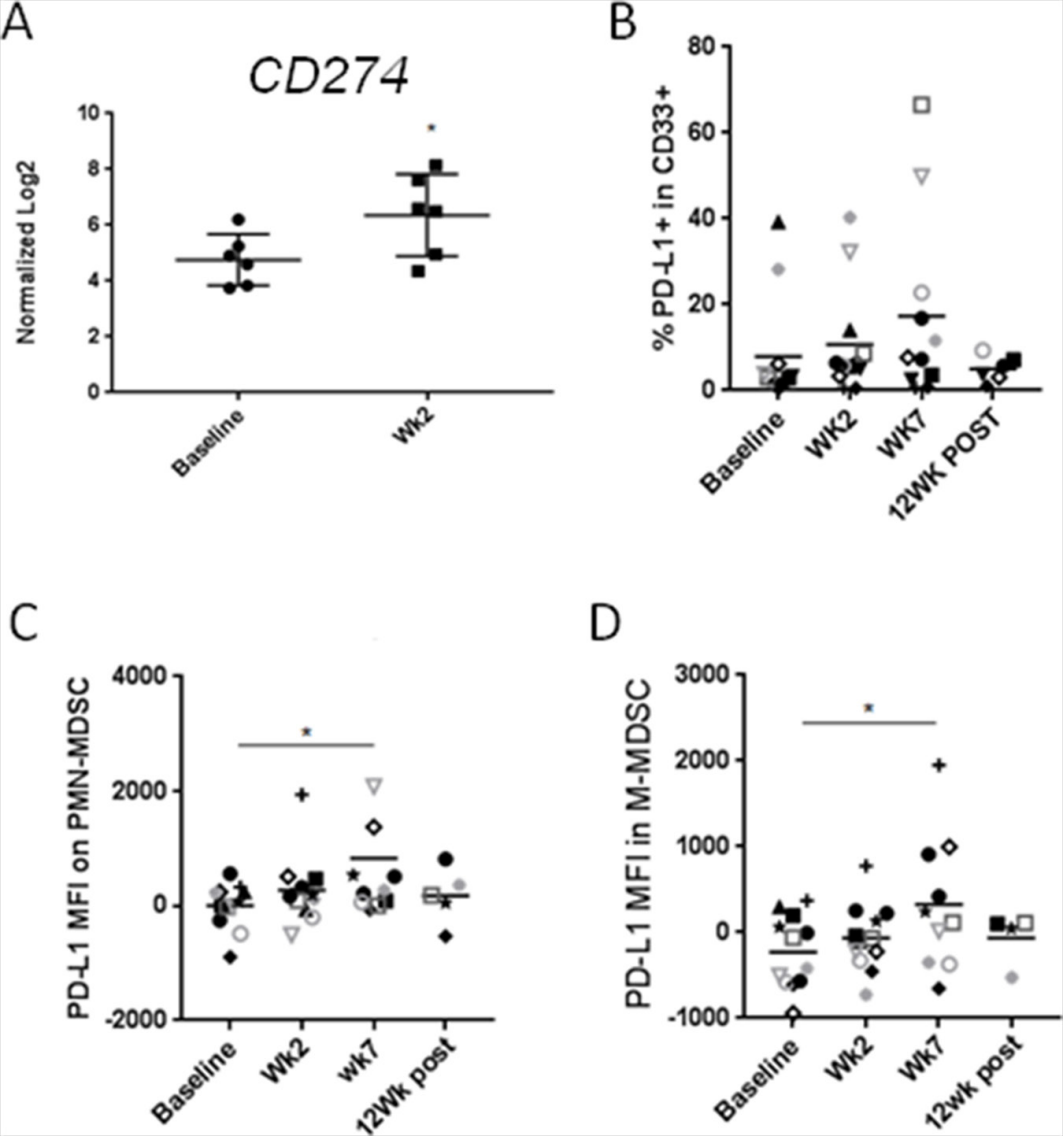
CMT results in the upregulation of PD-L1/CD274on HNSCC-associated MDSCs **(A)** mRNA expression levels of CD274 in PBMCs in patients undergoing CMT at the beginning and at the second week of therapy were compared using Nanostring analysis (*n* = 6). Protein expression level of CD274 on CD33^+^ myeloid cells **(B)**, PMN-MDSC **(C)**, and M-MDSC **(D)** were analyzed using flow cytometry. Paired student *t*-test; ^*^p<0.05, ^**^p<0.005, ^***^p<0.0005.

Blocking immune checkpoints such as PD1/PD-L1 has proven valuable strategy for treatment of various human cancers including HNSCC [24–27]. However, therapeutic efficacy of PD1/PD-L1 blockade critically depends on the expression level of PD-L1 in the tumor and in the tumor microenvironment. While, chemoradiation was recently found to upregulate PD1 expression on both CD8 ad CD4 T cells [28], the status of PD-L1 on cancer-associated myeloid cells, such as MDSCs, was uncharacterized. Based on our gene expression analysis, CMT resulted in significant upregulation of *PD-L1/CD274* expression on circulating PBMCs within the first two weeks (Figure 5A). We further examined protein levels of PD-L1 in various myeloid subsets using flow cytometry in HPV^+^ patients. As shown in Figure 5B, PD-L1 expression was gradually increased during CRT in CD33^+^ myeloid cells, reaching 2.5 fold increases at week 7 CMT (*p < 0.05*). Similarly, PD-L1 was significantly upregulated on the surface of both PMN-MDSC and M-MDSC at week 7 of CMT (Figure 5C and 5D, respectively). Altogether, our results may suggest that PD-L1 upregulation on circulating MDSC limits immunostimulatory effect of CMT.

## DISCUSSION

The long-term efficacy of cancer therapies, including radiation therapy, requires generation of systemic antitumor activity [29]. In spite of limited number of patients, our prospective biospecimen collection study in CMT-treated HPV^+^ HNSCC patients identified several therapy-induced effects on the immune system. The cytokine/chemokine profiling underscores the role of IL-6 as a proinflammatory cytokine secreted by myeloid cells, T cells as well as tumor cells upon inflammatory signals and as a result of therapeutic treatments such as CMT [30]. Importantly, IL-6 plays key role in promoting tumor progression and immunosuppression, mainly through the downstream activation of JAK/STAT3 signaling [31, 32]. STAT3 is an oncogenic transcription factor and a central immune checkpoint regulator, with a negative effect on antigen-presentation as well as T cell and NK cell activity [21, 22]. While our gene expression results remain only correlative, the elevated expression of *IL6R*, *STAT3* and its downstream target *CD274* in PBMCs from HNSCC patients, together with results of immunophenotyping of myeloid suppressor cells and the elevated arginase activity strongly suggest that CMT transiently stimulates immunosuppressive IL-6/STAT3 signaling. The effect of RT on the upregulation of PD-L1 expression on tumor cells have been previously described in mouse tumor models [33] and high levels of PD-L1 expression on cancer cells are known to be associated with radioresistance in HNSCC patients [34]. It has been reported that local irradiation of tumor upregulates expression of PD-L1 in tumor infiltrated myeloid cells in mouse tumor models [24], however, the systemic effect of RT on PD-L1 expression in HNSCC patients has been poorly understood. To our knowledge this is the first longitudinal study demonstrating upregulation of PD-L1 on circulating PMN- and M-MDSCs in HNSCC patients undergoing combinatorial RT. Together with growing evidence from preclinical and clinical studies [24–27], our observations highlight potential of targeting STAT3 and/or PD-L1 to improve CMT efficacy in HNSCC patients possibly by neutralizing this checkpoint molecule on immunosuppressive myeloid cells as well as cancer cells.

Beyond IL-6 activating STAT3 through IL-6R/JAKs, the CMT-treated patients showed upregulation of plasma levels of VEGF, which activates STAT3 through VEGFR1/2 tyrosine kinase domains [35–37]. VEGF plays key immunomodulatory roles in cancer patients by inhibiting maturation of dendritic cells and promoting accumulation of regulatory T cells and MDSC [38]. In contrast to our observation, Sridharan *et al.* recently reported decrease in VEGF level in HNSCC patients who received radiation to a dose of 70Gy concurrently with cisplatin [39]. The discrepancy may result from the greater number of low grade cancers included in this analysis, which also found the inverse correlation between tumor staging and the level of VEGF, with the highest VEGF levels in the T1-tumor stage patients. Nevertheless, this report suggested that decrease of VEGF is compensated by the upregulation of other proangiogenic mediators such as placental growth factor (PLGF) and angiopoietin-2 (ANG2). Both PLGF and ANG2 are well known downstream targets of JAK/STAT3 signaling [40–42]. Thus, findings from this report in general agree with our observations that CMT regimen can trigger activation of tolerogenic/angiogenic STAT3 in HNSCC patients. Finally, CMT elevated plasma levels of CCL2, which is a monocyte/MDSC chemoattractant promoting tumor progression and metastasis [43]. CCL2 induces accumulation and suppressive function of myeloid derived suppressive cells in tumor microenvironment in STAT3-dependent manner [44]. The increasing levels of tolerogenic IL-6, VEGF and CCL2 following CMT contrast with the increased levels of Th1 cytokines such as IFNγ or proinflammatory TNFɑ. However, as recently demonstrated by Wong *et al.* high levels of IFNγ together with TNFɑ can synergize to suppress Th1 response through upregulation of COX-2 [45]. In turn, COX-2 augments immunosuppressive functions of MDSC by promoting expression of indoleamine 2,3-dioxygenase (IDO), inducible nitric oxide synthase (iNOS/NOS2) and IL10 [46]. Consistently with this report, COX-2, IDO, NOS2 and IL10, as well as Th2 cytokines IL-4 and IL-5, were significantly increased in immune cells in HNSCC patients after two weeks of CMT. These potential tolerogenic effects correlate with reduction of IL-12 levels, which is likely to interfere with antigen-presentation and generation of antitumor immunity.

Overall, our observation suggests that CMT significantly affects the landscape of systemic immunity in HNSCC patients. Changes in genes expression and plasma cytokines and chemokines implicate that RT concurrent with systemic therapies can promote systemic immune suppression that possibly results in tumor recurrence and metastasis. This study provides rationale for combining inhibitors of IL-6/STAT3 and PD-1/PD-L1 signaling with CMT in order to augment therapeutic efficacy and durability of responses in patients with HNSCC and potentially other solid tumors.

## MATERIALS AND METHODS

### Clinical study design and specimen collection

The clinical protocol including the relevant informed consent form was approved by the institutional review board at City of Hope (IRB-14255), and the study was conducted in accordance with the amended Declaration of Helsinki and the International Conference on Harmonization Guidelines. The eligible patients were diagnosed with a lymph node-positive stage III/IV squamous cell carcinoma of head/neck and were required to receive standard-of-care radiation therapy (RT) with concurrent cisplatin or cetuximab (a monoclonal antibody against the epidermal growth factor receptor (EGFR)) treatments. The clinically and pathological involved lymph nodes were prescribed to receive the full dose of radiation. Blood samples were collected before or during the course of RT at week 1, 2, 4, 6, 7 and at 6 and 12 weeks after treatment completion (Supplementary Table 1). Radiation doses ranged from 66-70 Gy, given in 2-2.2 Gy fractions, using intensity modulated radiation therapy (IMRT) techniques.

Blood specimens were processed to isolate PBMCs and plasma as described previously [47]. Blood samples were collected in a BD Vacutainer CPT Tube. Upon receiving, the samples were centrifuged at 1800xg/RT (18°C) for 20 min using slow acceleration/deceleration. Plasma and PBMCs were collected separately and stored in −80°C or liquid nitrogen, respectively.

### Nanostring analysis

Patients’ peripheral blood was centrifuged in Vacutainer CPT tubes (BD) at 1,800×g/18°C for 20 min with slow acceleration/deceleration. PBMCs were suspended in CryoStor (BioLife Solutions/CS10) freezing media and stored in liquid nitrogen [47]. For gene expression analysis, mRNA was isolated from PBMCs from baseline and the second week draw during CMT from 4 HPV^+^ patients and 2 HPV^–^ patients (number 7, 8, 11, 12, 13, and 14) (Table 1) using mRNA isolation kit on Maxwell Rapid Sample Concentrator instrument (Promega). RNA quality was verified using the Bioanalyzer-2100 (Agilent). Gene expression was analyzed using Human PanCancer Immune Profiling panel (XT-CSO-HIP1-12) on the nCounter system (NanoString Technologies, WA) following manufacturer’s recommendations. Data analysis was done using nSolver 3.0 software (NanoString Technologies, WA). Raw data were normalized by stable housekeeping gene selected automatically by the system (Supplementary Table 6). Heat-maps and cell type profiling analysis were generated by the nSolver advanced analysis.

### Plasma biomarker assessment

Human cytokine, chemokine and growth factor concentrations were measured using Cytokine 30-plex Human Panel (ThermoFisher Scientific) run on the Luminex FLEXMAP 3D System at the Clinical Immunobiology Correlative Studies (CICSL) core at City of Hope. To assess the fold changes of serum level of each factor, the concentration was Log2-transformed and normalized to the baseline.

### Flow cytometry

For immunostaining of patients’ PBMC, the frozen cells were quickly thawed in 37°C water bath and washed. Cells were then re-suspended in media with 20% of matched plasma and incubated for at least 2 h at 37°C. Next, cells were washed with PBS with DNase and stained using Zombie UV viability dye (Biolegend), pre-incubated with CD16/32 antibody to block unspecific binding and then stained with fluorescently-labeled antibodies; CD14, CD33, CD15, and HLA-DR (Themofisher). For intracellular staining of pSTAT3, cells were fixed and permeabilized using Foxp3/Transcription Factor Staining Buffer Set (Themofisher) after surface staining. Cells were then stained with fluorescently-labeled antibodies specific to tyrosine 705-phosphorylated STAT3 (pSTAT3; BD Biosciences), Flow cytometric data were collected on BD Fortessa (BD Biosciences) and analyzed using FlowJo software (version 10.4.1, TreeStar).

### Arginase assay

Frozen plasma samples were thawed in 37°C and analyzed for arginase activity using Arginase Activity Assay Kit (Sigma) according to the manufacture’s protocol. Briefly, plasmas were centrifuged in membrane filter tubes (Amicon Ultra-0.5, Millipore Sigma) to deplete urea and incubated with Arginase buffer and substrate in 37°C for 2 h. Level of urea, a byproduct of Arginase enzymatic reaction, was measured using a chromogen that forms a colored complex specifically with urea and the optical density was read at 430 nm.

### Statistical analysis

Levels of plasma analytes were compared in longitudinally collected samples. We compared fold changes of cytokine/chemokines in comparison to levels in plasma samples collected at baseline. Changes of gene expressions between baseline and week 2-radiation in patient samples was analyzed from the normalized Log2 data from nSolver analysis of 6 patient samples. Paired student t-test was used for statistical analysis; *p*<0.05 was considered significant. All statistical analysis was computed using GraphPad Prism v 7.0.

## SUPPLEMENTARY MATERIALS TABLES


