# Supplementary material for: Combined modality radiation therapy promotes tolerogenic myeloid cell populations and STAT3-related gene expression in head and neck cancer patients
Source: Oncotarget. 2018 Feb 2;9(13):11279–90. doi: 10.18632/oncotarget.24397 (PMC5834279; doi:10.18632/oncotarget.24397)
Supplement: Supplementary file 1 [file oncotarget-09-11279-s001.pdf]

Combined modality radiation therapy promotes tolerogenic myeloid cell populations and STAT3-related gene expression in head and neck cancer patients

SUPPLEMENTARY MATERIALS

Supplementary Table 1: Protocol schema

Pre-treatment	Radiation with concurrent chemotherapy or cetuximab							Follow-up period	
	Week 1	Week 2	Week 3	Week 4	Week 5	Week 6	Week 7	Week 13	Week 19
B, N	B	B, N		B		B	B	B	B

Abbreviations: B, blood collection; N, lymph node biopsy.

Supplementary Table 2: Differential expression: week 2/baseline (Cytokine probe set)

Probe label	Log2 fold change	P-value
NOD2	1.31	0.000655
SPP1	4.07	0.00123
VEGFA	2.67	0.00264
IL2RB	−0.477	0.00343
IL6R	1.25	0.00393
JAK3	−1.13	0.00486
IL1RN	3.98	0.0049
IL12RB2	−0.795	0.00689
CCR5	1.35	0.00929
FLT3LG	−1	0.00956
TYK2	−0.701	0.00987
TNFSF14	2.05	0.0152
CCR1	1.62	0.0197
IL10	2.42	0.0221
IL8	2.22	0.0234
TNFSF8	1.42	0.0246
IFNL1	−0.519	0.03
HLA-DOB	−1.15	0.0303
TGFB1	0.8	0.0367
IL5	0.862	0.0393
LTB	−1.11	0.0397
IL4R	0.68	0.0404
CCR4	0.745	0.0408
IL1A	3.65	0.0446
IL1B	2.56	0.05

Supplementary Table 3: Differential expression: week 2/baseline (Chemokine probe set)

Gene	Log2 fold change	P-value
KLRB1	−1.01	0.000678
CX3CR1	−2.26	0.00237
CXCR2	−1.15	0.0032
TNFRSF1A	1.03	0.00336
TNFSF12	−1.23	0.00353
IL11RA	−1.17	0.00582
TNFRSF1B	1.19	0.00824
CCL7	6.01	0.00838
IL32	−1.75	0.00901
CCRL2	2.6	0.0113
CXCL16	0.956	0.0125
CCL2	5.35	0.0147
CCR3	2.3	0.0154
C1QBP	−0.578	0.0157
CCR1	1.62	0.0197
IFIT1	2.93	0.0208
STAT3	0.559	0.021
CXCL2	3.01	0.0232
IL8	2.22	0.0234
CCL20	3.68	0.0235
CXCL3	4.22	0.0265
CCL22	1.75	0.0407
CCR4	0.745	0.0408
CMKLR1	−1.06	0.0408
IFIT2	2.96	0.0446
CCR7	1	0.045
CCL8	2.79	0.0491
TNFSF15	2.24	0.0499

Supplementary Table 4: Differential expression: wk 2/baseline (T cell function probe set)

Gene	Log2 fold change	P-value
IL18RAP	−1.05	0.000281
TP53	−0.883	0.000321
EGR1	−1.61	0.00554
CD86	0.794	0.00617
IL12RB2	−0.795	0.00689
LCK	−1.44	0.00694
CCR5	1.35	0.00929
DPP4	−1.42	0.00941
TNFSF14	2.05	0.0152
CD3E	−1.34	0.0176
CD1C	−1.45	0.0184
CCR1	1.62	0.0197
CD27	−1.35	0.0244
TBX21	−0.825	0.0298
MS4A1	−1.06	0.0301
IL18R1	−0.787	0.0319
IL5	0.862	0.0393
IL4R	0.68	0.0404
CCR4	0.745	0.0408

Supplementary Table 5: Differential expression: Wk 2/baseline (NK cell function)

Gene	Log2 fold change	P-value
IL18RAP	−1.05	0.000281
KLRB1	−1.01	0.000678
KLRF1	−1.63	0.000734
IL12RB2	−0.795	0.00689
KLRK1	−0.839	0.0163
CCR1	1.62	0.0197
NCR1	−0.743	0.0214
KLRC2	−0.807	0.0246
KLRG1	−1.38	0.0309
IL18R1	−0.787	0.0319

Supplementary Table 6: House keeping genes used for normalization of gene expression

Gene Name
Eef1g
Ppia
Rpl19
Abcf1
Sf3a3
Polr2a
Gusb
Oaz1
Sdha
Nubp1
Sap130
Hdac3
Alas1
G6pdx
Tubb5
Eif2b4
Polr1b
Edc3
Hprt
Tbp
